# Moving knowledge into action for more effective practice, programmes and policy: protocol for a research programme on integrated knowledge translation

**DOI:** 10.1186/s13012-017-0700-y

**Published:** 2018-02-02

**Authors:** Ian D. Graham, Anita Kothari, Chris McCutcheon, On behalf of the Integrated Knowledge Translation Research Network Project Leads, Donna Angus, Davina Banner, Tracey Bucknall, Sandra Dunn, Michelle Gagnon, Wendy Gifford, Christina Godfrey, Bev Holmes, Tanya Horsley, Alison M. Hutchinson, Janet Jull, Susan Law, Martha MacLeod, Robert K. D. McLean, Kelly Mrklas, Tram Nguyen, Katrina Plamondon, Jo Rycroft-Malone, Shannon L. Sibbald, Kathryn M. Sibley, Dawn Stacey, David Kenneth Wright, Euson Yeung

**Affiliations:** 1 School of Epidemiology and Public Health, University of Ottawa, 307D- 600 Peter Morand Crescent, Ottawa, ON K1G 5Z3 Canada; 2 School of Health Studies, Western University, Health Sciences Building, Room 222, London, Ontario N6A 5B9 Canada; 3 Integrated Knowledge Translation Research Network, Centre for Practice-Changing Research, The Ottawa Hospital Research Institute, 501 Smyth Road, Box 711, Ottawa, Ontario K1H 8L6 Canada

**Keywords:** Integrated knowledge translation, Knowledge translation, Implementation, Collaborative research, Research co-production, Knowledge mobilization, Research use, Participatory research, Knowledge transfer

## Abstract

**Background:**

Health research is conducted with the expectation that it advances knowledge and eventually translates into improved health systems and population health. However, research findings are often caught in the know-do gap: they are not acted upon in a timely way or not applied at all. Integrated knowledge translation (IKT) is advanced as a way to increase the relevance, applicability and impact of research. With IKT, knowledge users work with researchers throughout the research process, starting with identification of the research question. Knowledge users represent those who would be able to use research results to inform their decisions (e.g. clinicians, managers, policy makers, patients/families and others). Stakeholders are increasingly interested in the idea that IKT generates greater and faster societal impact. Stakeholders are all those who are interested in the use of research results but may not necessarily use them for their own decision-making (e.g. governments, funders, researchers, health system managers and policy makers, patients and clinicians). Although IKT is broadly accepted, the actual research supporting it is limited and there is uncertainty about how best to conduct and support IKT. This paper presents a protocol for a programme of research testing the assumption that engaging the users of research in phases of its production leads to (a) greater appreciation of and capacity to use research; (b) the production of more relevant, useful and applicable research that results in greater impact; and (c) conditions under which it is more likely that research results will influence policy, managerial and clinical decision-making.

**Methods:**

The research programme will adopt an interdisciplinary, international, cross-sector approach, using multiple and mixed methods to reflect the complex and social nature of research partnerships. We will use ongoing and future natural IKT experiments as multiple cases to study IKT in depth, and we will take advantage of the team’s existing relationships with provincial, national and international organizations. Case studies will be retrospective and prospective, and the 7-year grant period will enable longitudinal studies. The initiation of partnerships, funding processes, the research lifecycle and then outcomes/impacts post project will be studied in real time. These living laboratories will also allow testing of strategies to improve the efficiency and effectiveness of the IKT approach.

**Discussion:**

This is the first interdisciplinary, systematic and programmatic research study on IKT. The research will provide scientific evidence on how to reliably and validly measure collaborative research partnerships and their impacts. The proposed research will build the science base for IKT, assess its relationship with research use and identify best practices and appropriate conditions for conducting IKT to achieve the greatest impact. It will also train and mentor the next generation of IKT researchers.

## Background

Health research is conducted with the expectation that it advances knowledge and eventually translates into improved health systems and population health. However, research findings are often caught in the know-do gap: they are not acted upon in a timely way or not applied at all. The failure to put research findings into action is therefore a major societal issue and contributes to the estimated $200B (USD) of wasted research funding because the full potential of research was not realized [1].

The magnitude of the know-do gap has stimulated governments and research funders around the globe to recognize the importance of the active translation of research into action [2]. Where historically the problem of research underutilization was considered simply a dissemination failure (knowledge users unaware of research), some now suggest this gap results from knowledge production failures (not producing research addressing knowledge user problems).

A widely recognized and accepted tenet of knowledge translation is the integration of knowledge users throughout the research process. Integrated knowledge translation (IKT) is advanced as a way to increase the relevance, applicability and impact of results [3, 4]. It shares common principles with many collaborative research approaches: co-production of knowledge, participatory research, linkage and exchange, Mode 2 knowledge production, engaged scholarship and community-based participatory research [5–10].

This approach proposes researcher/knowledge user collaboration as a key step in achieving societal impact and a way for society to speak to science. IKT shifts from a paradigm where the researcher is expert to one where researchers and knowledge users are both experts bringing complementary knowledge and skills to the team. They collaborate on issue-driven research with the expectation the research will generate implementable solutions to long-standing problems [11]. With IKT, the knowledge users work with researchers throughout the research process, starting with identification of the research question—they are actively engaged in the governance, priority setting and conduct of the research. Knowledge users represent all those who would be able to use research results to inform their decisions (clinicians, managers, policy makers, patients/families and others). Increasingly, stakeholders (governments, funders, researchers, health system managers and policy makers, patients and clinicians) are showing interest in the idea that IKT generates greater and faster societal impact. Stakeholders include all those with an interest in the issue or research, some of whom (knowledge users) are in a position to make direct use of the research in decision-making while other stakeholders are not but nevertheless want the issues and problems addressed.

Research funders have also been considering how to increase the impact of the research that they fund and their role in knowledge translation [2, 12–14]. ‘Integrated knowledge translation’ is a Canadian research funder innovation, initially advanced by the Canadian Health Services Research Foundation [6] and referred to as Knowledge Exchange in the late 1990s/early 2000s. The concept was adopted and refined at the Canadian Institutes of Health Research which coined the term integrated knowledge translation in the first decade of 2000s [15]. To promote the concept of partnered research, these organizations created funding opportunities that required collaboration between researchers and knowledge users. The Canadian Institutes of Health Research launched several funding initiatives promoting IKT (e.g. Partnerships for Health System Improvement, Knowledge Synthesis, Knowledge to Action, Community Based Research on HIV/IADs, Industry-Partnered Collaborative Research funding opportunities [16, 17] and Canadian Institutes of Health Research’s (CIHR’s) Strategy for Patient-Oriented Research competitions) [18]. Alberta Innovates (formerly Alberta Innovates: Health Solutions), a provincial research funding agency, initiated Partnership for Research and Health Innovation in the Health System and the Collaborative Research and Innovation Opportunities to encourage collaboration between researchers and knowledge users and to help translate research into improved health [19]. Similar funding competitions exist globally (of note is the Patient-Centered Outcomes Research Institute in the USA that only funds collaborative research) [20]. Funders have also created centres mandated to promote knowledge user engagement, accelerate research application and more efficiently harvest research benefits (e.g. Australian Academic Health Centres, Dutch Academic Collaborative Centres, UK Academic Health Science Centres, UK Collaborations for Leadership in Applied Health Research and Care—CLAHRC) [21–25]. Interest in this concept has also been demonstrated recently by publication of papers and commentaries on the topic in at least one journal [3, 26–31].

Although IKT is broadly accepted, the actual research supporting it is limited and there is uncertainty about how best to conduct and support IKT. A limited number of scoping, realist and other reviews suggest there is value of researchers and knowledge users working collaboratively and others are underway [32–35]. Emerging scholarship focusing on participatory action research [8, 36], the UK CLAHRCs [37–42] and CIHR’s evaluation of its IKT programmes [43, 44], is beginning to support the claims that IKT may lead to increased knowledge user capacity to use research; produce research that is more useful to knowledge users; increase the use of research in practice, health systems and policy decisions; and improve patient and health system outcomes. Studies are appearing describing how research partnerships work [45–47]. There is some evidence to suggest that in these collaborative research partnerships, researchers are the ones who benefit more by learning about the knowledge users’ context [11, 48]. Other studies reveal engagement of knowledge users can influence researchers’ approaches to research and the review of grants [49, 50]. However, the evidence is not yet conclusive on the impacts of IKT. At least one survey study failed to find an association between researcher/knowledge user engagement and research utility [51], suggesting that the factors determining effective IKT have yet to be clearly identified. Knowledge of IKT among researchers varies [52], and there is limited evidence about how researchers and knowledge users should go about collaborating. Despite the slim evidence base, stakeholder enthusiasm for IKT continues to grow. The expectation of enhanced impacts from IKT has seldom been critically assessed nor has the research partnering process been systematically studied. In response, Gagliardi and colleagues have recently suggested a research agenda for IKT [53].

The proposed research will build the science base for IKT, determine its effectiveness at increasing research use and identify best practices and appropriate conditions for conducting IKT to achieve the greatest impact on research use. The goals, objectives and outputs of the 7-year research programme are described in Table 1.

**Table 1:** Research programme goals, objectives and outputs

Goal 1: advance understanding of the concept of IKT from the perspectives of knowledge users, researchers, funders and universities Objectives: 1. Describe researcher and knowledge user partnerships and the conditions under which these partnerships succeed or fail. 2. Identify research funding mechanisms designed to support IKT and explore their effect on knowledge user engagement in research. 3. Identify how university (dis)incentives influence researcher involvement in IKT. Potential outputs: The knowledge generated will be immediately relevant to four groups: knowledge users and their organizations needing more relevant research, researchers wanting to do IKT, universities wanting to encourage IKT by faculty and funders wishing to make informed decisions about their policies and investments in support of IKT. The cumulative knowledge generated will fundamentally enhance our understanding of how and why researcher/knowledge user collaborations work and provide information on how to maximize the use of IKT as a strategy to address the underutilization of research.
Goal 2: assess the impacts of IKT Objectives: 1. Synthesize existing research on the benefits and challenges of IKT. 2. Determine what ways and under what conditions IKT adds value to research findings for knowledge users. 3. Generate new evidence on the outcomes and impacts of IKT. 4. Meta-synthesize the findings from all our research programme projects to identify the benefits, risks and implications of doing IKT and the circumstances under which IKT is most appropriate and impactful. Potential outputs: enhanced knowledge of the benefits, impacts, disadvantages and implications of IKT.
Goal 3: develop and adapt IKT theories and measurement tools Objectives: 1. Continue to evolve and test KT/implementation frameworks, particularly those incorporating IKT. 2. Develop and validate measures to assess partnering processes and impacts of IKT 3. Develop and test theory-based strategies/interventions designed to (a) support organizations’ meaningful engagement with researchers and use of research and (b) strengthen knowledge user organizational leadership for IKT. Potential outputs: further refinement of existing frameworks of IKT. Measures to reliably and validly assess IKT partnerships and their impacts. Better quantification of these phenomena for research purposes. Better research partnerships when partners use the measures to diagnose the quality of their relationships and identify challenges/issues that they can address to prevent relationship breakdown.
Goal 4: convert evidence gained from the research programme into accessible resources and build IKT research capacity to accelerate capturing the benefits of health research in Canada and abroad Objectives: 1. Develop IKT training manuals, tools, sessions for researchers and knowledge users. 2. Increase capacity for IKT among researchers and knowledge users. 3. Engage funding agencies, universities, healthcare organizations to develop, implement and evaluate strategies to support researcher/knowledge user engagement. Potential outputs: guidance documents/manuals and training sessions for both knowledge users and researchers on how to develop and nurture effective research partnerships. Researchers skilled in IKT (10–14 new HQP, > 100 trainees). Knowledge users at multiple levels (executives, managers, policy makers, clinicians) capable of participating in and using research. Effective strategies to promote IKT. A strengthened research community of practice on IKT. Academic incentives and funder policies better aligned to support IKT.

### Conceptual framework guiding the research programme

This research programme is informed by four main conceptual frameworks: (a) the Rycroft-Malone et al. framework for collaborative research (FCR) [38, 42], (b) the Research Impact Continuum (RIC) translational framework [54], (c) the Knowledge to Action framework (KTA) for implementation [55, 56] and (d) the Gifford model of leadership [57, 58].

The FCR identifies nine domains influencing knowledge use and impact stemming from researcher/knowledge user collaborations: knowledge and knowledge production, facilitation, patient and public involvement, knowledge sharing and exchange, geography, actors/agents, temporality, architecture of the knowledge user organization and its processes and context (the interconnecting and supporting relationships between all these domains). The RIC distinguishes between research and the practice of translation, highlights the role of research in translation, including IKT, and focuses attention on research impact. Indicators of success/impact guided by the RIC framework [54] include advances in knowledge (e.g. discoveries, publications), capacity enhancement (e.g. new HQP, trainees, researchers, knowledge users with IKT skills), health system and policy impacts (e.g. use of programme findings in decision-making). The KTA framework highlights the interplay between knowledge creation and application and identifies key components required for planned action. The Gifford leadership framework specifies the leadership and management behaviors that positively influence knowledge translation, including relation-oriented behaviors (supporting, developing, recognising others), change-oriented behaviors (visioning, providing direction, building coalitions) and task-oriented behaviors (clarifying roles, monitoring and procuring resources).

## Methods/design

The approach to this Canadian Institutes of Health Research 7-year foundation grant is interdisciplinary and cross-sectoral, using multiple and mixed methods that best reflect the complex and social nature of research partnerships. Knowledge users are full members on the research programme and individual project teams and will continue to be actively involved in every step of the research process. To allow the research programme to be more inclusive than those listed on this proposal, the programme is organized as a researcher/knowledge user network known as the Integrated Knowledge Translation Research Network: Doing Research with the People Who Use it (https://iktrn.ohri.ca).The network has been specifically designed to include researchers interested in IKT who include early career, mid-career and senior researchers (referred to as IKT experts, currently *n* = 40); IKT trainees (currently *n* = 16), knowledge user experts from research funding agencies; charities; health services and health authorities and other organizations (currently *n* = 31); and a methods resource group comprising knowledge translation and implementation science experts (currently *n* = 11). When feasible to do so, we will use an IKT approach within the projects to expand the team’s experiential knowledge of IKT mechanisms. All projects are guided by programme goals and objectives. Table 2 presents the programme work streams along with their objectives, rationale, research questions, level of partnerships and outputs.

**Table 2:** Project descriptions, corresponding programme objective, outputs

Project	Level of partnership	Outputs	Contribution to goal/objectives
Knowledge syntheses Objective: to provide the network’s research with a strong foundation based on the existing literature. Rationale: while IKT has not been extensively studied, there are relevant theories, models and frameworks; primary studies on the process of IKT; and information on how IKT can be measured, but this knowledge has yet to be synthesized in any systematic way. All knowledge syntheses will be registered in appropriate registries.			
1a: Comparing and contrasting integrated knowledge translation with five approaches often used in knowledge translation: identifying and synthesizing germinal literature and consulting with experts Question: What are the similarities and differences between IKT, participatory research, engaged scholarship, co-production of knowledge, Mode 2 knowledge production? Design: concept analysis Sample: germinal articles/books identified by key informants Project lead: Nguyen, Graham	N/A	Conceptual clarity on concepts	Goal 3—objective 1
1b: Systematic review of research engagement frameworks Question: (1) What research engagement frameworks exist? (2) What are the similarities and differences in engagement framework attributes? Design: systematic review and analysis of framework Sample: published and grey literature Project lead: Jull, Graham	N/A	Repository of research engagement frameworks Meta-engagement framework	Goal 3—objective 1
1c: Understanding when integrated knowledge translation works: a realist review Questions: (1) How can IKT process be theorized and evaluated? (2) What are the impacts (positive/negative) of IKT? (3) What are the mechanisms by which IKT produces impacts under what conditions? Design: realist review methods [59] Sample: published and grey literature Project lead: Kothari, Horsley*	Partnership level will be included in analysis but expect focus on researchers and research user (manager/clinician) partnerships	Evidence of effectiveness/impact Factors contributing to IKT success/failure Mechanisms by which IKT works under what conditions	Goal 1—objectives 1,2 Goal 2—objectives 1,2,3
1d: Guideline dissemination and implementation interventions for nursing: a systematic review Question: (1) To identify and assess the effects of the interventions employed to increase the use of practice guidelines in nursing? (2) To what extent has IKT been used as a strategy to increase guideline uptake and to what effect? Design: systematic review of the published evidence Sample: all rigorously designed randomized controlled trials of the effectiveness of strategies to influence the uptake of practice guidelines by nurses (MEDLINE, EMBASE, CINAHL, AMED, PsychINFO) Project lead: Godfrey, Graham	N/A	Evidence on the effectiveness of strategies (including IKT) to increase use of guidelines in nursing	Goal 1—objective 1 Goal 2—objectives 2,3
1e: What are the available tools to evaluate the partnering process in healthcare research? A scoping review Question: (1) What are the existing measures, assessments, and tools that evaluate researchers’ and stakeholders’ role, satisfaction, expectation, and contribution in healthcare research partnerships? Design: scoping review [60, 61] Sample: MEDLINE, PsyINFO, Embase, CINAHL, grey literature (government reports, websites, etc.). Project lead: Nguyen, Graham	Researcher and knowledge user (research projects)	Inventory of instruments	Goal 3—objective 2
Case studies Objective: to amass evidence on the process of IKT and its impact Rationale: given that an experimental study design will never be feasible or practical to use to determine the effectiveness of integrated knowledge translation or to understand how it works, other study designs are required. We have elected to conduct a number of retrospective and prospective case studies to learn how IKT works with what effect.			
2a: An evaluation of an academic-health services partnership network Question: (1) How does the partnership between the Deakin University Centre for Quality and Patient Safety Research and Victorian Health Services work? (2) What are the impacts of such relationships? (3) What are the benefits and issues professors and health service leaders in these partnerships face? Design: retrospective case study [62–65], key informant interviews Sample: 6 partnerships Project lead: Bucknall, Hutchinson	University and health service partnerships	How does IKTR work when the partnership is at the level of a school and health service Impacts from IKT	Goal 1—objectives 1,2,3 Goal 2—objective 3 Goal 3—objective 1
2b: Link between knowledge user participation and impact: the case of BORN Ontario Questions: (1) How does BORN Ontario engage knowledge user organizations in co-production of Data Dashboards when not all organizations can be included on committees? (2) How do organizations without committee representation perceive their engagement with BORN? (3) Are there differences in uptake of the Dashboards among organizations that are and are not represented on committees? (4) What criteria should be used to select knowledge user representatives when all research users cannot be included? Design: retrospective and prospective case study [62–65] Sample: BORN and 10–20 stakeholder organizations Project lead: Dunn	Knowledge producer organization and knowledge user organization (BORN-Ontario hospitals)	How IKT works when both partners are organizations How IKT works when not all the user partners can be engaged because of their numbers Factors contributing to IKT success/failure Role of leadership in IKT Perspective of health/funder organizations on IKT Impacts of partnerships	Goal 1—objective 1 Goal 2—objective 3 Goal 3—objective 1
2c: Exploring the role of leadership and other factors in creating and sustaining a university-health authority partnership for knowledge creation, knowledge translation and organizational change Question: (1) What is the role of leadership in developing and maintaining the partnership? (2) How does leadership at different levels of the partnering organizations influence the relationship and the outcomes of the partnership? Design: secondary analysis of a longitudinal-processual analysis of a case study of two organizations; interviews with key informants [66] Sample: Northern Health-University of Northern British Columbia partnership Project lead: MacLeod, Gifford	University and regional health authority partnership	How IKT works when partners are an academic centre and regional health authority Factors contributing to IKT success/failure Role of leadership in IKT Impacts of partnership	Goal 1—objective 1
Experiential IKT knowledge Objective: to learn about IKT from doing it. Rationale: given that IKT is as relatively recent phenomenon and that little knowledge has been codified about how to do IKT, we believe much can be learned from those using this approach. We will be eliciting case stories from network members (researchers and knowledge users) about their experiences working in an IKT way. To focus discussion and research in the field, we will be generating a number of concept papers that identify areas in need of greater conceptualization or research.			
3a. IKT Casebook Question: What can we learn from researcher and knowledge user experiences using an IKT approach? Design: secondary analyses of case stories. Sample: network members Project lead: Graham, Kothari, McCutcheon, Gagnon*	All levels	IKT casebooks over the 7 years of funding Identification of factors Related to successful and unsuccessful partnerships	Goal 1—objective 1 Goal 2—objectives 2,3
3b. Concept papers Question: What are the issues that should be tackled to advance the science of IKT? Design: theoretical, conceptual or review papers Sample: network members Project lead: Graham, Kothari, McCutcheon, Angus*	All levels	Manuscripts highlighting gaps in IKT science	Goal 3—objective 1
Funder-focused studies Objective: to understand IKT and its impact by studying funded studies that required the use of an IKT approach. Rationale: important sources of data on integrated KT are studies funded through IKT funding opportunities that require knowledge user partnerships. Much can be learned from these studies about how IKT was operationalized, how the research was conducted, the experiences of researchers and knowledge users working in partnership and the results of the study. These IKT studies will also be used to study the effectiveness of these studies by identifying and analyzing their effects and impacts. Given our many knowledge user partners are research funders, we intend to exploit opportunities to identify funded IKT studies so that we may study them.			
4a: Advancing the science of integrated knowledge translation with health researchers and knowledge users: understanding current and developing recommendations for integrated KT practice Questions: (1) What is known about how IKT is conducted and to what effect? (2) What IKT methods have been used in funded projects with what impact? (3) What IKT methods, metrics and evaluation methods are recommended by individuals who are knowledgeable and have a vested interest in IKT? Design: explanatory mixed methods approach including: scoping review, web-survey and interviews, nominal group consensus Sample: published and grey literature, principle investigators of CIHR funded IKT grants, IKT researchers and knowledge users Project lead: Sibley, (funder, clinician, patient knowledge users) Additional funding being sought for the project.	Researchers-knowledge user partnerships	Strategies for doing IKT Evidence of IKT impact Recommendations about how to conduct and measure IKT	Goal 1—objective 1 Goal 2—objectives 1,2,3 Goal 4—objective 1
4b: Health research funder strategies to promote IKT and their effectiveness Question: (1) How do major funders around the globe support IKT? (2) How have funders evaluated the effectiveness/impact of their investments in IKT? (3) How have funder IKT strategies evolved overtime? Design: scan of website analysis and interviews with research funders-replication of previous funder scans [2, 43, 44] Sample: ~ 30 research funding agencies from around the world Project lead: Graham, doctoral student McLean, Holmes*	Researcher and knowledge user (research projects)	List of how funders incentivize IKT research Synthesis of funder evidence for their IKT funding programs Changes in funder approaches to IKT over a 15-year period	Goal 1—objective 2 Goal 2—objective 3 Goal 3—objective 1
4c: Impact of CIHR IKT funding opportunities. Questions: (1) What is the relationship between extent of knowledge engagement on iKT grants and impact on addressing CIHR’s mandate areas? Design: secondary quantitative analysis of CIHR’s KT Programme evaluation data (multiple regression) [43, 44] Sample: web-survey data from researchers and knowledge-users in the open and IKT funding opportunities Project lead: Graham, McLean, Rycroft-Malone* (funder knowledge user)	Researcher and clinician, manager, policy maker research user partnerships (CIHR PHSI, Knowledge to Action and Knowledge Synthesis grants)	Evidence on the relationship between research-user engagement and outcomes attributed to research grants	Goal 1—objective 2 Goal 2—objectives 1,3
4d: Researchers’ and knowledge users’ perceptions of research partnerships Question: (1) What makes a successful partnership? (2) What are barriers and facilitators to successful partnerships? (3) How can funding agencies facilitate required partnerships? Design: secondary analysis of web-based survey of CIHR researchers and knowledge users. Sample: CIHR researchers and knowledge users surveyed in 2009. Project lead: Sibbald, Graham	Researcher and knowledge user (research projects)	Factors related to successful partnerships Identified barriers and facilitators of partnerships Recommendations for funders interested in supporting IKT research	Goal 1—objective 1, 2 Goal 2—objective 2
Organization-focused studies Objective: to describe the experiences of IKT from the perspective of knowledge user organizations Rationale: much of the literature on researcher/knowledge user partnerships has focused on the individual who is the knowledge user partner rather than the organization that individual. Our assumption is that one of the next frontiers in IKT will focus on organizations as knowledge user partners so it is imperative to understand the partnering experiences and expectations of knowledge user organizations.			
5a: Understanding IKT from the perspective of organizations: a mixed methods study Questions: (1) How do organizations make decisions about who and when they should partner with researchers? (2) What is the organizational experience of researcher partnerships? Design: survey, content-analysis, focus-group interviews Sample: organizations listed on CIHR funded research projects as knowledge users. Project lead: Horsley	All 3 levels	Knowledge of perspectives and experiences with IKT process Strategies used by organizations to manage IKT Guidance on how organizations attract and work with researchers to do research on the organization’s priority areas	Goal 1—objectives 1,2 Goal 2—objectives 2,3 Goal 3—objective 1 Goal 4—objective 1
5b: Principles of partnering with researchers to inform substantial health system change Questions: (1) How do health authorities make decisions about which researchers to partner with and when? (2) What is the health authority experience with researcher partnerships? (3) What policies and processes do health authorities have to guide research partnership decisions? Design: website document review, web-survey, qualitative methods-interviews Sample: Canadian regional health authorities. Project lead: Botting	Researchers and health authority partnerships	Health authority perspectives and experiences with IKT processes Strategies used by organizations to manage IKT Guidance on how health authorities manage research proactively and reactively partnerships with researchers	Goal 1—objectives 1,2 Goal 2—objectives 2,3 Goal 3—objective 1 Goal 4—objective 1
Researcher and university-focused studies Objective: to understand the implications of using an IKT approach for researchers. Rationale: researchers report that universities tend not to incentivize researchers to do IKT and that performance metrics seldom value partnerships with knowledge users. For example, impact citation metrics are highly valued where efforts to develop, nurture and sustain partnerships with knowledge users may not be. Few data actually exist on how university performance expectations support or discourage IKT. These data could be used to highlight university barriers to IKT and encourage discussion of these barriers.			
6a: Are health researchers involved in research focused on uptake of research into practice reporting research translation and impact activities on their CVs? A web-survey of researchers Question: How do researchers report their KT and IKT activities in their CVs? Design: web-survey. Sample: researchers on mailing list of the international Knowledge Utilization Colloquium and the Collaborative Healthcare Improvement Partnership (CHIPs) theme group of the Canadian Association for Health Services and Policy Research. Project lead: Stacey	N/A	Knowledge about researcher CV practices	Goal 4—objective 1
6b: University Schools of Nursing policies promoting or discouraging IKT Question: What university incentives and policies affect researcher involvement in IKT? Design: document analyses and key informant interviews Sample: university tenure and promotion criteria, university administrators Project lead: Banner	N/A	List of Canadian SON, and other university policies and their influence on IKT	Goal 1—objective 3
Measurement studies Objective: to develop, refine and validate measures to assess partnering processes and impacts of IKT Rationale: currently, validate measures of IKT partnering and measures of the impacts of IKT are lacking. Without being able to measure how well a researcher/ knowledge user partnership is functioning or the outcomes of doing IKT, the ability to develop strategies to make partnerships more effective or to demonstrate the effectiveness of IKT will remain limited.			
7: Developing and testing a valid, sensitive, reliable IKT questionnaire based on the Kothari et al. indicators [67, 68] Design: quantitative methods—web-survey design and psychometric testing, qualitative methods—cognitive interviewing Sample: emergent case studies Project lead: Kothari, Graham	N/A	Measures of partnership process and impact Predictors of successful partnerships	Goal 3—objective 2
IKT tools and capacity building Objective: to develop IKT tools and training materials for researchers and knowledge users and to increase researchers’ and knowledge users’ capacity for IKT. Rationale: the use of IKT will not increase unless researchers and knowledge users acquire knowledge about IKT and the skills to partner and work together. Building this capacity will be even more needed should studies demonstrate the value of IKT.			
8a: Knowledge translation plan guides: a pragmatic, conceptual synthesis Question: What guidance is provided to researchers and research-users about doing KT and IKT? Design: systematic review and document analyses Sample: funder websites, peer reviewed and grey literature Project lead: McCutcheon, doctoral student Mrklas	N/A	Repository of KT and IKT guidance documents Analysis comparing documents to facilitate decisions about usefulness of each	Goal 4—objective 1
8b: Development and field testing of IKT training modules for researchers and knowledge users: identifying competencies for integrated knowledge translation Question: (1) What are IKT competencies for researchers (students and early career) and knowledge users? (2) What are researchers’ and knowledge users’ preferred instructional strategies to learn IKT competencies? (3) How effective are IKT training modules? Design: literature review, key informant interviews, Delphi methods, pre/post evaluation Sample: convenience sample of researchers and knowledge users, network members Project lead: Yeung, doctoral students Plamondon and Mrklas (knowledge users)	N/A	List of IKT competencies Training modules	Goal 4—objectives 1,2
8c: Conducting patient-oriented research: an online tutorial for the use of a collaborative framework for community-research partnerships Question: (1) What should be the guiding features for researcher-indigenous community research partnerships? (2) What guidance should be provided to researchers and indigenous partners about working in an IKT way? Design: theory and literature based, user testing Sample: an advisory group of indigenous people Project lead: postdoc Jull, Graham, indigenous knowledge users Funding by the Ontario Strategy for Patient-Oriented Research (SPOR) Support Unit	Researcher-knowledge user (indigenous patients and communities)	Training module	Goal 4—objectives 1,2
8d: Experiences of patient-researcher partnerships in integrated knowledge translation: a study to develop online training for patient-oriented research Question: (1) What are patient and researchers experiences as research partners? (2) What guidance should be provided to researchers and knowledge-users about doing KT and IKT? Design: qualitative methods—interviews, pedagogical theory Sample: 15 patients and 15 researchers Project lead: Law, Wright, Graham Funding by the Ontario SPOR Support Unit	Researcher-knowledge user (patient)	Understanding of the experiences of patients and researchers of patient-researcher partnerships Two scalable training modules for patients and researchers on how to manage patient-researcher partnerships	Goal 4—objectives 1,2
Emergent projects during 2nd half of grant - Emergent partnership case study - Emergent partnership case study - Developing and testing theory-based strategies/interventions to increase knowledge user partnering with researches and to increase capacity to use research - Other emergent studies	Depends on the case TBD	How IKT works or does not work Factors contributing to IKT success/failure Role of leadership in IKT Impacts of partnerships Strategies proven effective at increasing organizations’ capacity to partner with researchers and to use research findings	Goal 1—objectives 1,2 Goal 2—objective 3 Goal 3—objectives 1,2 Goal 3—objectives 1,2 Goal 3—objective 3 TBD
Project: Meta-synthesis of program projects findings	All 3 levels	Science and practice of IKT greatly advanced Canada recognized as a leader in studying IKT	Has potential to address objectives from goals 1–3

*IKT* integrated knowledge translation, *CIHR* Canadian Institutes of Health Research

*Knowledge user

Several knowledge syntheses are proposed to increase understanding of the concept of IKT (projects 1a–b), how IKT works and with what impact (projects 1c–d) and to identify tools to evaluate the partnering process (project 1e). A novel aspect of the research is that three initial multiple-case studies (projects 2a–c) anchor the programme during the first half of the grant. The case studies are both retrospective and prospective and will provide data and knowledge on how IKT works, its impact and the degree of engagement required to optimise impact. The case studies will provide insight into IKT partnerships at two levels:

- Inter-organizational: partnerships between BORN (Better Outcomes Registry and Network) Ontario and hospitals providing maternity care (project 2b);
- Regional: partnerships between Deakin University Centre for Quality and Patient Safety Research and health services in the State of Victoria, Australia (project 2a); university and regional health authority partnership (UNBC and Northern Health) (project 2c).

The case studies will be complemented with other projects focusing on other aspects of IKT and other programme objectives. For example, project 3a is intended to capture the network members’ and organizations’ experiential knowledge about working in an IKT way while project 3b is about network members reflecting on the field and identifying where the science should focus. Several studies focus on funder programmes to promote research undertaken using an IKT approach (projects 4a–d). Other studies focus on the perspective of an organization that becomes the partner in an IKT project (projects 5a–b) or the perspective of the researcher or university (projects 6a–b). Project 7 is designed to develop and test an IKT questionnaire. Finally, projects 8a–d are about IKT tools and developing training modules for researchers and knowledge users. We anticipate that research questions generated from projects will subsequently be embedded into future case studies as this will be an efficient way to study these topics without having to launch new stand-alone studies.

More case studies will be added as the grant proceeds. Several knowledge user partners are already identifying opportunities to study IKT ‘in the field’, and the project structure enables timely incorporation of these opportunities into the programme. In years 2–3 of the grant, future case studies will be initiated. Initial criteria for selecting new projects will include addressing knowledge gaps identified in the programme’s ongoing studies, prioritization of future studies by knowledge users and the Advisory Committee and feasibility. Towards year 4, intervention studies will be launched to test theory-based strategies to improve research partnering and build health organization capacity for research partnering and research use.

Finally, a meta-synthesis of findings from all projects will be completed to discern patterns and differences between different knowledge user groups (patients, clinicians, managers, policy makers), organizations (healthcare delivery institutions, health authorities, ministries of health, health research funders) and contexts and to develop materials to facilitate IKT and uptake of the findings. Team members are very interested in executives/managers, who have great potential to activate organizational change for research-informed decision-making but are understudied.

### Training

Objective 2 of goal 4 is about creating a training environment for IKT research and supervising and mentoring graduate students and postdoctoral trainees and colleagues. To achieve this objective, the programme has an innovative and bold plan. We have incorporated funding to support one postdoc, two PhD and two master’s students a year. This will produce five to seven master’s, two to three PhDs and three to four postdocs over the life of the grant with expertise in the science and art of IKT. The CIHR KT evaluation [44] revealed that IKT projects are more likely to develop more highly qualified personnel per $100k grant than a grant of the same value in the open competition ([15], Table 2 p6). Given the value of producing the next generation of KT scholars, we have also included student/trainee stipends to facilitate the involvement of students in as many of our projects and professional networks as possible. Over the life of the grant, this represents 35–40 studentships. We will also develop short internet training modules on various aspects of IKT for researchers and knowledge users.

The programme will also fund one to two researcher/knowledge user internships/year (eight over the course of the grant). These will be for graduate students and postdoctoral trainees to spend 3 months sharing their research expertise with one of our knowledge user organization partners while they learn about policy making in the real world. This is an efficient way for trainees to learn about policy making while at the same time exposing the organizations to researchers in training. The internship programme will be modeled after CIHR’s Science-Policy Fellowships developed by IDG when he was at CIHR (https://www.canada.ca/en/health-canada/services/science-research/career-resources/fellowship-programs/science-policy-fellowships-program.html, Accessed 22 Dec 2017). Interns will be assigned both an academic and policy maker mentor.

#### Governance and strategies to reduce risk to the research programme

A governance structure is in place to ensure an enduring focus on excellence, flexibility and ability to capitalize on emerging opportunities and help the programme remain on track. An Executive Committee, chaired by a Program Leader (Scientific Director—IDG), will be responsible for day-to-day operations. It will include the Deputy Scientific Director (AK), two researchers, two knowledge users, one trainee and one research associate. Sub-committees responsible for science (IKT theory, methods and measures), impact (network performance monitoring) and training will provide leadership in these areas. The impact committee will convene an impact workshop with the project leaders to produce a logic model or theory of change for the network and determine how to collect data to test it. An international Advisory Committee (AC) comprising knowledge users and IKT experts will provide guidance on all aspects of the programme, annually review the performance of programme projects and suggest strategies to reduce risk of bias in study design, data analysis and interpretation. Terms of reference for all committees will be finalized in collaboration with members and reviewed annually. The research programme team will use a collaborative decision-making approach.

We have designed the programme so that no one project carries all the intellectual weight of the programme putting the programme at risk should it fail. The breadth, nature and number of projects is one risk-mitigating strategy—the whole is greater than the sum of its parts. Other strategies to ensure success including a formalized process for prioritization, peer-review, optimization of quality and monitoring project progress will be developed to ensure only the best, most relevant projects are advanced. Each project will be required to have a written proposal which will be reviewed by the Advisory Committee in terms of its relevance to the programme’s objectives; potential to generate new knowledge, study design and methods; potential to achieve the intended outcomes/impact; and resources required.

Monthly team teleconferences and one annual face-to-face meeting will maintain team cohesiveness and momentum and facilitate knowledge sharing. Team meetings, along with the annual review of projects by the Advisory Committee, will identify challenges faced by projects and marshal the collective wisdom of the team and/or Advisory Committee to overcome them. The diversity of the team and the richness of its content and scientific and knowledge user expertise will be a considerable asset for finding mitigating strategies.

#### Research programme limitations

The most significant limitation relates to the initial use of observational and quasi-experimental study designs. Given the focus on research partnerships, we expect that researchers and knowledge users will not be sympathetic or agreeable to experimental study designs that would require being randomized to use an IKT approach. However, to maximize overall scientific rigor, the research programme will rely on mixed methods and triangulation of findings and strive to select the most rigorous study designs for individual projects. For example, the use of both retrospective and prospective case studies is preferable to using only retrospective case studies. Another example is that we will be studying the influence of funded IKT studies by comparing the resulting impacts with the impacts of curiosity-driven research (essentially a non-randomized control group). We also anticipate that rigor will be increased by including projects that involve different types of knowledge users (e.g. patients, indigenous groups, clinicians, health services decision-makers, funders, etc.) and examine different levels of partnership (e.g. project, health authority, etc.). These settings will allow us to describe dominant patterns across varied arrangements, thereby enhancing the generalisability of the work. During the course of the 7-year lifespan of the programme, we also expect to build on the lessons learned from the first wave of studies and propose and conduct more rigorous and methodologically innovate projects in subsequent waves. We also anticipate that in future prospective case studies, we will be able to introduce and evaluate interventions to improve partnering.

#### Knowledge translation

Our KT strategies consist of two approaches: IKT and end-of-project KT/knowledge mobilization. In keeping with the focus on integrated knowledge translation, the Integrated Knowledge Translation Research Network will use an IKT approach in all of its studies to ensure the projects address the issues of concern of our knowledge user partners and hopefully produce useful findings that can be acted upon by our and other knowledge users.

Our end of project KT activities will be guided by the CIHR’s Guide to Knowledge Translation Planning [69]. For academic audiences, we will produce peer-reviewed journal articles for relevant journals. For knowledge user and stakeholder audiences, we will use a number of strategies to disseminate our work. To facilitate dissemination, we will create a website for the network that will house all the tools and products we produce. We will create a web blog that will serve as a vehicle for early dissemination of findings, engaging the public and cross-fertilizing our ideas with each other and scientists in other areas. We will use social media (e.g. Twitter) to create a presence of the IKT Research Network. We will also use a newsletter to inform audiences about our activities and to disseminate findings.

The suite of training materials, tools and sessions described above will be available online to help researchers and knowledge users build their capacity to engage in IKT. Another Network KT strategy for disseminating findings and capacity building in IKT will be the hosting of a bi-annual symposium on the State of the Art and Science of IKT. The symposium may occur around an annual meeting of the Canadian Association of Health Services and Policy Research (CAHSPR), KT Canada’s annual general meeting or another conference. The purpose of these symposia will be to create a forum for knowledge users and researchers to share their experiences with partnering, present findings from the latest research on how best to undertake collaborative research, explore opportunities for working together/network development and offer skill-building seminars and workshops on doing IKT, strategies for effective research partnerships and maintaining relationships.

We also intend to host events similar to the CIHR Best Brains Exchanges [70] with the National Alliance for Provincial Health Research Organizations (NAPHRO), the Health Charities Coalition of Canada, health sector organizations and the Canadian research Tri-Councils (Canadian Institutes of Health Research, Social Sciences and Humanities Research Council, National Science and Engineering Research Council) around the research programme findings. These exchanges will bring together researchers and policy makers/administrators in a relaxed and confidential environment to discuss IKT research and its policy implications. We will include trainees in these events so they can learn how they work, how to host them and to make connections with policy makers, health system managers and funders.

## Discussion

We have proposed the first interdisciplinary, systematic and programmatic research endeavor and network focusing on IKT. The research programme was developed and will be executed with knowledge user organization executives, managers, policy makers, clinicians and patients. We will ground the programme in knowledge generated through systematic, scoping and realist reviews. Taking advantage of our pre-existing productive relationships with provincial, national and international organizations, we will use ongoing and future natural IKT experiments as multiple case studies in order to study IKT in depth. Case studies will be retrospective and prospective as the 7-year grant timeline will enable us to undertake prospective longitudinal studies of IKT. We will study, in real time, the initiation of partnerships, funding processes, the research lifecycle and then outcomes/impact post project. In the latter years of the programme, we anticipate that these living laboratories [71] will also facilitate testing of strategies to improve the efficiency and effectiveness of the IKT approach. The research will also provide scientific evidence on how to reliably and validly measure collaborative research partnerships and their impacts. Built into the programme is a vibrant training and mentoring environment for trainees and researchers interested in the science of IKT and its application.

By conducting a meta-synthesis of multiple case studies and other strategic studies undertaken during the early years of the programme, we will be able to demonstrate how IKT works, under which circumstances and with which knowledge user groups. We will determine what IKT can and cannot do and learn how researchers and knowledge users develop and maintain research partnerships. When available, we will assess the impact of IKT on health system and patient outcomes. We will also ascertain how to promote IKT among knowledge users/knowledge user organizations and researchers. Significant potential and timely opportunities exist for improving how IKT is practiced and supported. By better understanding IKT, developing instruments to measure it and its impact, and designing effective strategies that support IKT, we will be positioned to improve knowledge translation and more thoroughly reap the benefits of research.

## Abbreviations

AC: Advisory Committee
CIHR: Canadian Institutes of Health Research
CLARHC: Collaborations for Leadership in Applied Health Research and Care
CV: Curriculum vitae
FCR: Framework for collaborative research
HQP: Highly qualified personnel
IKT: Integrated knowledge translation
KT: Knowledge translation
KTA: Knowledge to action
RIC: Research Impact Continuum
